# Preharvest Application of Commercial Products Based on Chitosan, Phosphoric Acid Plus Micronutrients, and Orange Essential Oil on Postharvest Quality and Gray Mold Infections of Strawberry

**DOI:** 10.3390/ijms232415472

**Published:** 2022-12-07

**Authors:** Razieh Rajestary, Panayiota Xylia, Antonios Chrysargyris, Gianfranco Romanazzi, Nikolaos Tzortzakis

**Affiliations:** 1 Department of Agricultural Food and Environmental Sciences, Marche Polytechnic University, Via Brecce Bianche, 60131 Ancona, Italy; 2 Department of Agricultural Sciences, Biotechnology and Food Science, Cyprus University of Technology, Limassol 3603, Cyprus

**Keywords:** chitosan, fruit quality parameters, sweet orange essential oil, phosphoric acid, preharvest treatments

## Abstract

Strawberry is a perishable fruit with a limited shelf life after harvest due to deterioration of quality and the development of gray mold, Rhizopus rot and other minor diseases. In this study, the effectiveness of commercial compounds based on chitosan, phosphoric acid plus micronutrients, and sweet orange essential oil (EO) in reducing decay and optimizing the quality of strawberries was analyzed. The plant canopy of a greenhouse crop was sprayed once and strawberry fruit were harvested three days later. Gray mold infections were evaluated after chilled storage for seven days at 4 ± 0.5 °C followed by five days shelf life. The qualitative parameters were recorded at harvest (initial day) and after three days of storage at room temperature (RT, 20 °C) or after cold storage and shelf life (CS, 4 °C). The application of sweet orange EO increased the antioxidant and flavonoid content at harvest, while a decrease was reported following three days of storage at RT. At the same time, increased ethylene production and weight loss were observed during CS three days after harvesting. Chitosan treatment maintained the harvest fruit quality and was effective in the control of postharvest decay. Our results suggest that the investigated natural compounds could improve strawberry quality after harvest. Since chitosan performed best in terms of maintaining quality and reducing postharvest decay, it could be considered as a good substitute for chemical-synthetic fungicides for the preservation of strawberry postharvest gray mold.

## 1. Introduction

Consumers adore strawberries (*Fragaria × ananassa* Duch.) for their distinct flavor, taste, and health benefits. Strawberries include a lot of bioactive components, including vitamins, amino acids, polyphenols, natural antioxidants, and anthocyanins [1]. A diet rich in antioxidants can prevent and minimize the risk of cardiovascular disease, cancer, and diabetes [2]. However, strawberry has a limited shelf life after harvest because of its susceptibility to fungal pathogens [3].

In the field, strawberry fruit can be infected with a fungus that can spread to nearby fruit during the postharvest period [4,5]. The primary fungal disease associated with strawberries while they are in storage is *Botrytis cinerea*, which is the cause of gray mold. Until now, the first line of defense against gray mold in strawberries in conventional agriculture has been the use of synthetic fungicides and preservation chemicals. However, when their use became more restricted due to legislation, pathogen resistance, and consumer concern over food safety and sustainability, research began to focus on alternate methods. One of the most promising alternatives for preventing fruit infections and delaying harvested product senescence in this context was determined to be the usage of natural bioactive substances [5,6,7,8,9,10].

Among the natural products investigated for their suitability for pre- and postharvest applications for the preservation of fresh commodities, edible coatings seem to be gaining more and more attention [11]. The increasing interest in these compounds derives from their ability to form a protective membrane that can serve as a barrier, controlling and/or decreasing the respiration and transpiration of the produce, thereby minimizing moisture loss [12]. In addition, edible coatings of various compositions present antimicrobial properties (antibacterial and antifungal), resulting in a decrease of postharvest decay [13]. In addition, plant-derived extracts, including essential oils (EOs), have been assessed for pre- and postharvest applications on fresh produce due to the antibacterial and antifungal properties that they possess [8,9,14,15,16,17,18,19,20,21].

The use of edible coatings with highly active antimicrobial ingredients, such as chitosan or EOs with multicomponent compositions, was recommended for reducing the potential for pathogen development [22,23,24,25,26,27]. Several studies have assessed the antifungal effectiveness of various EOs or chitosan on *B. cinerea*, examining the abilities of these compounds to stimulate plant defenses or form a film on fruit [28,29,30,31]. Moreover, exposure to EOs and chitosan has been shown to enhance the preservation of strawberry fruit quality, exhibiting increased concentrations of phenolics, anthocyanins, and flavonoids [15,32,33].

The objectives of the present study were to: (i) assess the efficacy of natural substances in preventing strawberry fruit postharvest degradation and gray mold development via preharvest greenhouse application of commercial products based on chitosan, phosphoric acid plus micronutrients and sweet orange EO; and (ii) to examine the impacts of these treatments on fruit qualitative parameters.

## 2. Results

### 2.1. Decay Evaluation

Chitosan was found to be the most effective compound to reduce gray mold (Figure S1) after 5 d shelf life, with a McKinney’s index of 36.19% and a 35% reduction in comparison to the control (Table 1). No significant difference was observed in the McKinney index between fruit treated with PA+MN and sweet orange (*Citrus* × *aurantium* L.) EO and the control at 5 d shelf life, although a tendency toward decay reduction was observed.

### 2.2. Fruit Quality

#### 2.2.1. Effects at Harvest Time

Preharvest use of chitosan, PA+MN, and sweet orange EO affected fruit quality parameters on the initial day of the quality experiment (three days after spraying and prior storage), as presented in Table 2. A reduction in fresh strawberry weight with chitosan compared to the non-treated (control) fruits was observed on the day of harvesting. The application of chitosan resulted in significantly lower titratable acidity (TA) (0.64%) in comparison to sweet orange EO treated and untreated (control) fruit (0.93 and 0.93%, respectively). Additionally, the ascorbic acid (AA) content of strawberries was found to be decreased with all three applied products compared to the non-treated fruits (control). According to the results from the ferric reducing antioxidant power (FRAP) assay, the antioxidant activity in strawberry fruit treated with sweet orange EO was higher than in the control (5.14 and 3.53 g kg^−1^ trolox, respectively) on Day 0. In contrast, the 2,2′-azino-bis(3-ethylbenzothiazoline-6-sulphonic acid (ABTS) assay showed that fruit treated with PA+MN and sweet orange EO had decreased antioxidants (7.19 and 7.46 g kg^−1^ trolox, respectively) compared to chitosan treatment and control (10.51 and 10.34 g kg^−1^ trolox, respectively). Among phenolic compounds, the flavonoid content in fruit treated with sweet orange EO (1.25 g kg^−1^ rutin) and PA+MN (1.07 g kg^−1^ rutin) was found to be increased compared to the control (0.66 g kg^−1^ rutin). Moreover, chitosan resulted in significantly decreased flavonoids compared to sweet orange EO treatment (0.80 and 1.25 g kg^−1^ rutin, respectively). Interestingly, the other examined parameters did not yield significantly different results between the applied treatments and the control (Table 2).

#### 2.2.2. Effects after Storage

**Figure 1:**
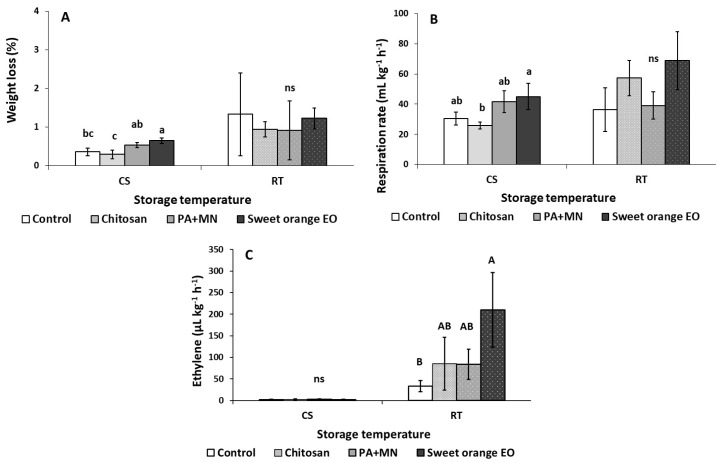
Impact of chitosan, phosphoric acid plus micronutrients (PA+MN), and sweet orange EO treatment on (**A**) weight loss (%), (**B**) respiration rate, and (**C**) ethylene production of strawberry fruit after three days of storage at RT (20 °C) and CS (4 °C). Error bars represent the standard deviation of the mean of three/six replicates. Treatments indicated with different letters for each storage condition differed significantly (Tukey’s honestly significant difference, at *p* ≤ 0.05), ns: not significant.

**Figure 2:**
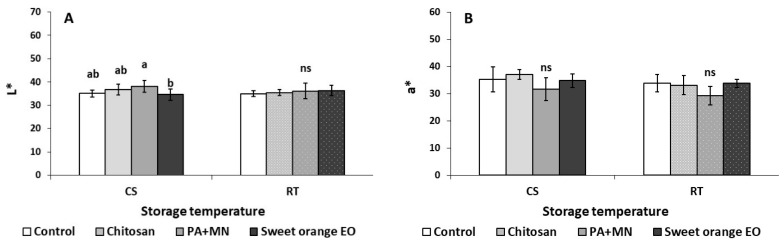

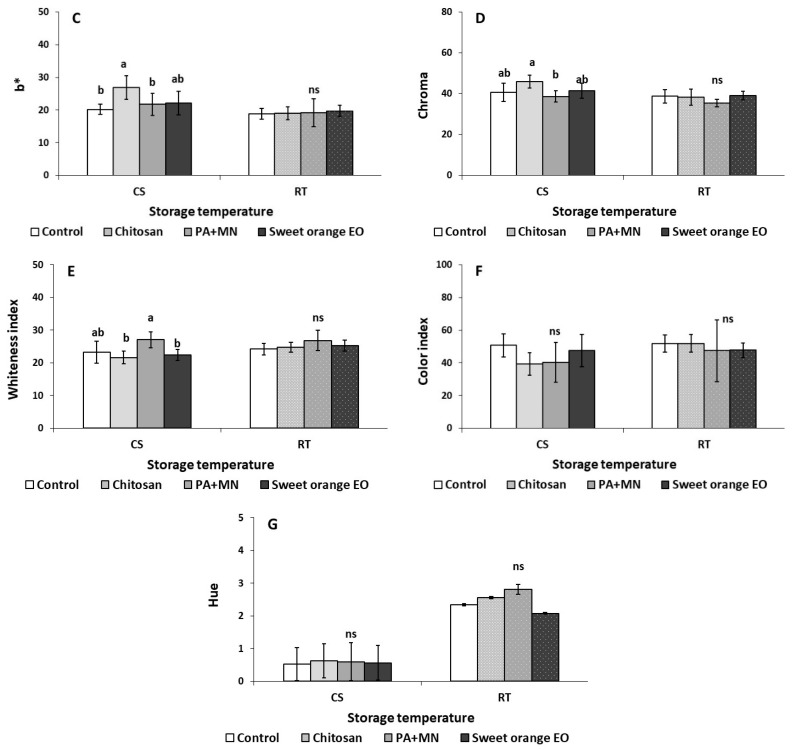
Effect of chitosan, phosphoric acid plus micronutrients (PA+MN), and sweet orange EO treatment and three days of storage at RT (20 °C) and CS (4 °C) on strawberry color parameters: (**A**) L* color, (**B**) a* color, (**C**) b* color, (**D**) Chroma value, (**E**) Whiteness index, (**F**) Color index, and (**G**) Hue angle. Error bars represent the standard deviation of the mean of three/six replicates. Treatments indicated with different letters for each storage condition differed significantly (Tukey’s honestly significant difference, at *p* ≤ 0.05); ns: not significant.

**Figure 3:**
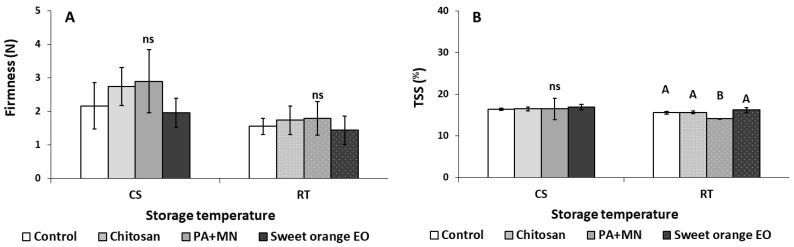

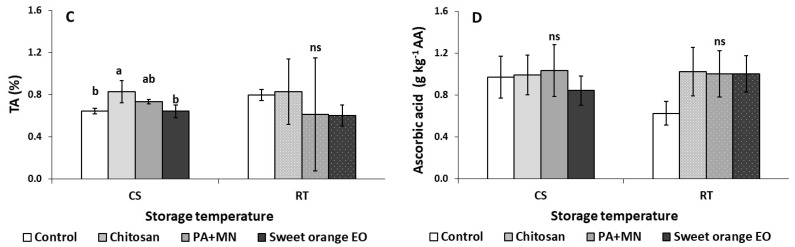
Effect of chitosan, phosphoric acid plus micronutrients (PA+MN), and sweet orange EO treatment on (**A**) firmness, (**B**) total soluble solids (TSS), (**C**) total acidity (TA), and (**D**) ascorbic acid (AA) of strawberry fruit after three days of storage at RT (20 °C) and CS (4 °C). Error bars represent the standard deviation of the mean of (three/six fruit) replicates. Treatments indicated with different letters for each storage condition differed significantly (Tukey’s honestly significant difference, at *p* ≤ 0.05); ns: not significant.

**Figure 4:**
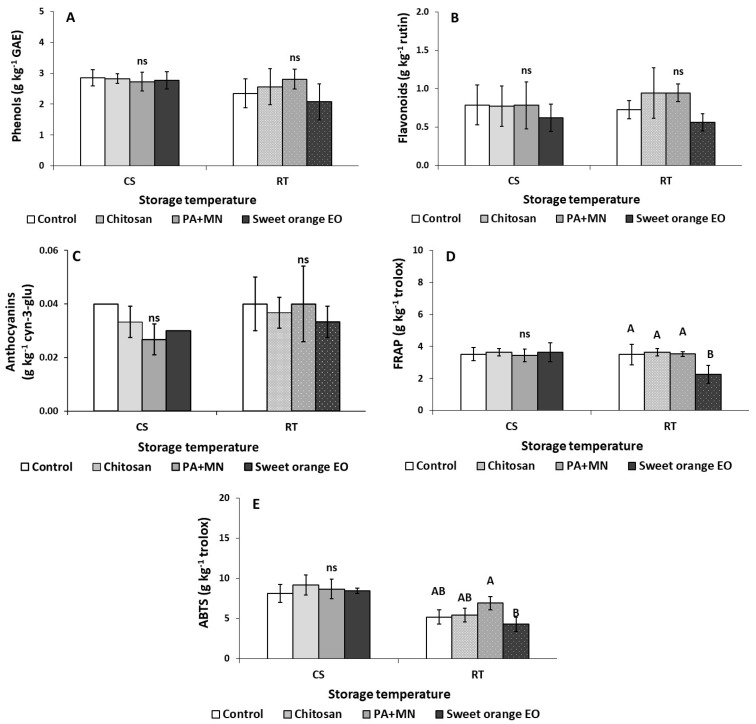
Effect of chitosan, phosphoric acid plus micronutrients (PA+MN), and sweet orange EO treatment and three days of storage at RT (20 °C) and CS (4 °C) on (**A**) total phenols, (**B**) total flavonoids, (**C**) anthocyanins, and (**D**,**E**) antioxidants (FRAP, ABTS). Error bars represent the standard deviation of the mean of (three/six fruit) replicates. Treatments indicated with different letters for each storage condition differed significantly (Tukey’s test significant difference, at *p* ≤ 0.05); ns: not significant.

The effects of the applied preharvest treatments on the quality attributes of strawberry fruit after storage for three days under different storage conditions (RT-20 °C and CS-4 °C) are presented in Figure 1, Figure 2, Figure 3 and Figure 4. After three days of storage at RT (20 °C), ethylene production in strawberry fruit treated with sweet orange EO increased (170 μL kg^−1^ h^−1^ ethylene) compared to control (non-treated fruits) (33.15 μL kg^−1^ h^−1^ ethylene) (Figure 1C). Total soluble solids (TSS) in fruit treated with PA+MN were lower (14.11%) than in all the other applied treatments (Figure 3B). The application of sweet orange EO was found to decrease the antioxidant content of strawberry fruit (FRAP: 2.26 g kg^−1^ trolox) in comparison to all other treatments, whereas the ABTS assay showed that PA+MN was able to increase fruit antioxidant content (6.92 g kg^−1^ trolox), as opposed to sweet orange EO (4.29 g kg^−1^ trolox), during three days of storage at 20 °C (Figure 4D,E). Interestingly, the other examined parameters did not yield significantly different results between the applied treatments and the control.

As illustrated in Figure 1A, after three days of CS (4 °C), the weight loss in fruit treated with sweet orange EO was higher (0.65%) compared to the control and chitosan treated group (0.36 and 0.30%, respectively). In addition, the application of PA+MN increased the weight loss (0.54%) in comparison to chitosan (0.30%). As shown in Figure 1B, sweet orange EO application yielded an increase of fruit respiration rates compared to chitosan after storage for three days at CS (45.00 and 25.78 mL kg^−1^ h^−1^ CO_2_, respectively). The application of PA+MN was found to increase the L* color value (38.22) of strawberry fruit, as opposed to sweet orange EO (34.60), after three days of storage at CS (Figure 2A). The b* color value increased with the application of chitosan (26.91) compared to PA+MN treatment and non-treated fruit (21.75 and 20.18, respectively) (Figure 2C). Chitosan was found to increase the chroma value of strawberry fruit (45.93) after three days of storage at 4 °C in comparison to PA+MN (38.62) (Figure 2C). The whiteness index (WI) increased with the application of PA+MN (27.06), as opposed to chitosan and sweet orange EO (21.63 and 22.38, respectively) (Figure 2C). Regarding the TA in strawberry fruit, treatment with chitosan resulted in an increased value (0.83%) compared to control and sweet orange EO (0.65 and 0.64%, respectively) (Figure 3C). No differences were reported between the treatments and the control for all other tested parameters (Figure 2, Figure 3 and Figure 4).

## 3. Discussion

The pre- and postharvest use of many natural compounds (including chitosan, phosphoric acid plus micronutrients, and essential oils) has been investigated in an effort to decrease the use of chemical fungicides and control the development of postharvest diseases [9,16,33,34,35]. In the present study, preharvest application of chitosan was found to significantly reduce the development of gray mold on strawberry fruit cv. ‘Festival’ during shelf life. This compound interferes with *B. cinerea*, slowing gray mold infection, since this natural biopolymer possess film-forming, antimicrobial, and eliciting properties [36,37]. Chitosan has been previously shown to be responsible for decreasing the spore germination or lysis of *B. cinerea* mycelium following preharvest application [38,39]. For example, a previous study demonstrated that the use of chitosan (1% *w*/*v* Chito Plant) among other natural compounds significantly reduced postharvest decay of sweet cherries in vivo and, at the same time, reduced the growth of decay causing fungi in vitro [34]. However, in the present study, differently from chitosan, PA+MN and sweet orange EO were found to be ineffective in controlling gray mold development on strawberry during storage conditions, even though they have the potential to express eliciting (PA+MN) and antimicrobial (sweet orange EO) activities toward gray mold [34]. These results can be ascribed to the ability of *B. cinerea* to survive at a latent stage in plant tissues and to its ability to develop at cold storage temperatures [40].

Regarding how preharvest treatments affect strawberry fruit quality before harvest and after three days of storage under various conditions (RT-20 °C and CS-4 °C), sweet orange EO treatment was found to have a greater impact on strawberry characteristics on the first day of application and during storage at 20 and 4 °C, compared to the other applied treatments. Significant weight loss (moisture loss) of strawberry fruit was observed with CS three days after storage following sweet orange EO and PA+MN application, in contrast to chitosan application. This might be due to the difference in composition of the applied products and especially the presence of chitosan. It is well known that chitosan can form a semi-permeable coating around fresh produce; this allows gaseous exchanges and minimizes fruit weight loss [33]. With these observations, the assumption that the application of chitosan can result in lower postharvest weight loss can be made.

The influence of chitosan on the control of weight loss was a vital factor with a great impact on the appearance of strawberry fruit, attributed to the formation of an additional layer by the natural compounds coating the surface stomata. This resulted in a reduction of transpiration against oxygen, carbon dioxide, and ethylene levels and dehydration, thereby limiting weight loss [41,42,43]. These results revealed that the firmness stability (as a symbol of high quality, related to pectin and other cell wall components) was maintained. Additionally, the presence of anthocyanin in fruit epidermis and cortex related to the red pigment did not change after coating with all compounds [44,45]. Our work confirmed that edible coatings served as barriers, preventing moisture loss, water transfer, and weight loss. This barrier also decreased the ability of the strawberries to absorb oxygen, which, in turn, delayed the rates of respiration and the associated loss of weight from the fruit’s surface. This finding was in agreement with the result of other studies [46,47]. Interestingly, after three days of CS, sweet orange EO was found to increase the respiration rate of strawberry fruits compared to chitosan treatment. Strawberries, as perishable fruit, tend to present a high respiration rate and are affected by storage conditions (i.e., temperature and duration) [16]. Sweet orange EO was reported to have fungicidal and insecticidal properties [48]. A previous study showed a decrease in the respiration rate of strawberry fruits following the postharvest application of 2% citrus (lemon, orange and mandarin) EOs during storage at 2 °C [16]. The findings in our study are not in accordance with that study; this might be attributed to the lower EO concentration applied (0.08% vs. 2%), the use of cold storage, and the time of application (pre- vs. postharvest), as well as the volatile nature of EOs, that could have evaporated prior to harvesting and/or during storage. Moreover, sweet orange EO, if used at higher concentrations on strawberry canopies, may induce phytotoxic effects.

Strawberries are non-climacteric fruit that can produce ethylene in limited amounts [49]. After storage at RT (20 °C) for three days, fruit treated with sweet orange EO presented increased ethylene production in comparison to non-treated fruit (control). Ethylene is elaborated in a number of processes besides ripening, including responses to pathogens and wounding, senescence of leaves, and the responses of various abiotic and biotic stress factors [50,51]. This result could be connected with the molecular characteristics of sweet orange EO, as volatile components might affect fruit quality parameters [16,52].

One of the key characteristics that influences consumer purchasing decisions is the bright red color of strawberry fruit. In the current study, the preharvest application of chitosan following three days of storage at 4 °C resulted in an increased chroma value and b* color value compared to PA+MN, indicating a darker red color in the fruit. According to a previous study, strawberry fruits treated with chitosan (1%) prior to harvest were able to retain their dark red color [33]. The results from the present work are in agreement with these observations; this could be correlated with the fact that no significant differences in total flavonoid content were reported, with flavonoids being the main pigments responsible for strawberry fruit’s color (including anthocyanins). Chitosan is approved as a basic substance for plant protection, and several applications are ongoing, both in experimental and practical applications, with the potential to replace synthetic fungicides in some settings [53].

The application of chitosan resulted in decreased TA of strawberry fruit three days after preharvest application, compared to sweet orange EO and control, whereas after three days of storage at CS, the opposite effect was observed. Interestingly, a minor impact of strawberry quality was observed with chitosan-based compounds, except for an increase in TA, which is known to influence the microbiological stability of fruit and phenolic compound content [54,55]. This could be attributed to the combination of chitosan application and cold storage (4 °C), as, in a previous study, it was shown that a chitosan coating and storage at 10 °C for six days delayed the ripening, decay, and senescing of strawberry fruit [42]. Fruit becomes sweeter as a result of an increase in TSS and a decrease in TA throughout the ripening process. In a prior study, an eco-product made of rosemary and eucalyptus EOs, applied preharvest to tomatoes, yielded biostimulant properties, increasing the ripening metabolism of the fruit while lowering firmness and the phenol and antioxidant contents [20]. The same EO-based product was further applied postharvest to tomato fruits (dipping and vapor application); it was found that this product was able to maintain the fruit’s quality attributes during 14 d of storage at 11 °C [21]. These previous observations, together with the results of the current investigation (the application of treatment based on sweet orange EO), suggest that EOs could be considered for the preservation of fresh fruits, with either pre- or postharvest application.

Strawberries are a good source of antioxidants; including polyphenols, vitamins (especially vitamin C), and flavonoids (i.e., anthocyanins), which makes them an important part of a diet that can minimize the oxidative stress related with chronic diseases [56]. In our study, the preharvest application of sweet orange EO was found to increase the antioxidant and flavonoid contents in strawberry fruits during harvesting time (three days after preharvest application), while following three days of storage at 20 °C (RT), a reduction in antioxidants was reported with the use of sweet orange EO. In a previous study, the postharvest application of citrus EOs to strawberry fruit resulted in an increase in antioxidants in the fruit, even after 4 d of storage at 2 °C [16]. The increase in antioxidants might be associated with the fact that EOs consist of a mixture of compounds that present antioxidant activities, while the loss of antioxidants during storage at RT might be connected to the evaporation of these EOs at higher storage temperatures, as well to storage duration.

Overall, the preharvest use of natural compounds such as chitosan and EOs on strawberries for the improvement of their postharvest quality and to prevent fruit decay is very encouraging. However, it is important to further investigate the type and time of application, the concentrations used, as well as the postharvest conditions during storage.

## 4. Materials and Methods

### 4.1. Plant Material and Growth Conditions

The cover material of strawberry plants (*Fragaria × ananassa* Duch, cv. ‘Festival’), grown in a commercial greenhouse, consisted of transparent polyethylene sheets. Experiments were undertaken in Kolossi, Limassol (Cyprus). Common cultivation practices were applied during the study: plants were fertigated (Plant Prod 20-20-20; Plant Prod 18-9-27, related to the percentage of N-P-K in the form of N-P_2_O_5_-K_2_O) using an irrigation system at the beginning of, and later in, the crop season. Additionally, azoxystrobin for *Sphaerotheca macularis* f. sp. *fragariae* and emamectin benzoate for worm and orange oil were applied once via spraying. The soil type was considered silt loam (6.75% clay, 39.21% sand and 53.98% silt) with 3.03% organic matter, a pH of 7.93, an electrical conductivity (EC) of 6.58 mS cm^−1^, and a calcium carbonate content of 21.67%. A completely randomized design was set up and six-month-old strawberry plant were used for this experiment.

### 4.2. Preharvest Treatments

Three different commercial products based on chitosan (Chito Plant, ChiPro GmbH, Bremen, Germany), phosphoric acid plus micronutrients (PA+MN) (KiForce, Alba Milagro, Milano, Italy), and sweet orange EO (Prev-Am Plus, Nufarm, Milano, Italy) were tested, resulting in four treatments, namely, (i) control, (ii) chitosan (1%), (iii) phosphoric acid plus micronutrients (PA+MN) (1%), and (iv) sweet orange EO (0.08%). The preparation of the solutions involved dissolving them in distilled water and stirring for 60 min. The control was distilled water. For each preharvest treatment, 90 plants were selected. Freshly prepared solutions were applied once by spraying 1.5 L of each compound throughout the plants with a mechanical mist sprayer. Fruit was harvested when it reached the commercial ripening stage based on the typical color (red skin) and shape, three days after spraying. For each treatment, 200 strawberries were counted at the picking date. Small fruit with defects were discarded. Strawberries were transferred to the laboratory within 1 h of picking for further experiments. Treated strawberries without visual damage which were homogeneous in color and size were selected and placed into small containers (1 L capacity).

### 4.3. Decay Evaluation

For each treatment, 42 fruit were tested (six for each of the seven replicates). Strawberry fruits were cold stored (CS) (4 °C) for one week and then exposed to five days of shelf life at 20 ± 1 °C. Strawberries were examined every day for decay symptoms and fungal growth (i.e., *B. cinerea*), and the number of infected fruit (disease incidence, expressed as a percentage) was recorded. According to a five-degree empirical scale, disease severity was recorded as follows: 0—fruit that is healthy; 1—fruit that is surface-infected between 1% and 20%; 2—fruit surface-infected between 21% and 40%; 3—fruit surface-infected between 41% and 60%; 4—fruit surface-infected between 61% and 80%; and 5—fruit that is surface-infected and sporulating at ≥81% [40]. The infection index, also known as the McKinney index, was represented as a percentage of the highest possible level and incorporates both the incidence and severity of the deterioration [57].

### 4.4. Quality Evaluation

According to each foliar application, strawberries were randomly selected, labeled, and weighed for fruit quality assessments (three replications with six fruits). Fruit was examined at harvest (initial day) and following three days of storage at room temperature (20 ± 1 °C) (RT) or shelf life (4 ± 0.5 °C plus 7 d at RT) (CS) with 95% relative humidity (RH). The physical and chemical properties of the fruit that were measured (on the initial and last day) included weight loss, color, firmness, rates of respiration, rates of ethylene production, total soluble solids-TSS, titratable acidity-TA, ascorbic acid-AA, anthocyanin content, total phenolics content, total flavonoid content, and antioxidant capacity. To this end, two different protocols were used: ferric reducing antioxidant power-FRAP and 2,2′-azino-bis3-ethylbenzothiazoline-6-sulfonic acid-ABTS.

#### 4.4.1. Weight Loss, Respiration Rate, and Ethylene Production

The weight difference of strawberries between the initial day and the third day was measured, and the results were expressed as weight loss percentage (%) = [(m_0_ − m_3_)/m_0_] × 100, where m_0_ is the initial weight and m_3_ is the weight after three days of storage.

The rates of respiration of the strawberries were analyzed by employing an electronic dual gas analyzer, (GCS 250 Analyzer, International Control Analyzer Ltd., Kent, UK); the results are expressed as mL of carbon dioxide (CO_2_) produced per kg of strawberry fresh weight per hour (mL kg^−1^ h^−1^ CO_2_). After being taken out of cold storage (4 °C), strawberries were placed on a lab bench for about one hour to warm to room temperature (20 °C). Fruit was then placed in a 1 L capacity container. A 1 mm diameter hole was drilled into the lead of the container and sealed with a tape to prevent gas flow. In order to stop gas from leaking from the packaging, gaseous samples were taken through septa with a syringe. Strawberries of known fresh weight and volume were considered for calculations of respiration rates, as follows: Respiration rate (mL kg^−1^ h^−1^ CO_2_) = (V_1_ − V_2_) × %CO_2_ *10/W × T where, V_1_ = Volume of container (L), V_2_ = Volume of fruit (L), W = Weight of fruit (kg), T = Time of enclosure (h).

The ethylene production of the strawberries was analyzed using an ethylene analyzer (ICA 56 Analyzer, International Control Analyzer Ltd., Kent, UK). Following respiration measurement, gaseous samples were tested for ethylene production. Ethylene rate was expressed as μL of ethylene produced per kg of strawberry fresh weight per hour (μL kg^−1^ h^−1^ ethylene).

#### 4.4.2. Color Evaluation

A Konica Minolta colorimeter (Chroma Meter CR 400 Konica Minolta, Japan) was employed to measure the surface color of the samples on days 0 and 3 (two values in each replicate/three replicates in each treatment). Fruit color was assessed by measuring the values of L* (lightness), a* (greenness [−] to redness [+]) and b* (blueness [−] to yellowness [+]). The chroma value (C), whiteness index (WI), color index (CI), and hue angle (h) were computed according to the following equations: $C={\left(a^{*2}+b^{*2}\right)}^{1/2}$, $WI=100-{\lfloor \left(100-L^{*}\right)}^{2}+a^{*2}+b^{*2}\rfloor {}^{1/2}$, $C=\left(a^{*}\times 1000\right)/\left(L^{*}\times b^{*}\right)$ and $h=\tan^{-1}\left(a^{*}/b^{*}\right)$, respectively [58,59].

#### 4.4.3. Firmness, Total Soluble Solids, Titratable Acidity, and Ascorbic Acid

Strawberry fruit firmness was measured by a texture analyzer (TA-XT plus, Stable Micro Systems, Surrey, UK) equipped with a 3 mm diameter cylinder stainless probe. The probe penetrating depth was 7 mm and the cross-head speed of the texture analyzer was 1 mm s^−1^. The results were reported as the maximum peak force in Newton (N) using the Texture Exponent 32 program (Stable Micro Systems, Surrey, UK).

The total soluble solids content of storage strawberry fruit was estimated using a digital pocket refractometer (Atago, Tokyo, Japan). Fruit was homogenized with a blender for 2 min before being filtered through muslin cloth for squeezing fruit juice. A few drops of the extract were placed on the refractometer prism glass and data were recorded through direct reading. Results of TSS were expressed as percentage (%).

Titratable acidity was analyzed using Mettler Toledo Food and Beverage Titrator (Mettler Toledo DL22, Mettler-Toledo AG, Schwerzenbach, Switzerland), as described by Tzortzakis [60]. Homogenized strawberry juice (5 mL) was placed in a 50 mL volumetric flask and the desired volume was obtained with distilled water. The extract was then titrated with 0.1 N NaOH (pH 8.2). The results are reported as citric acid percentage (%).

The ascorbic acid content was calculated according to the 2,6-dichlorophenolindophenol titration method [61]. Briefly, 5 g strawberry was extracted with 50 mL of 3% meta-phosphoric acid solution. The upper aqueous phase was then recovered by centrifugation at 3000× *g* for 15 min at 25 °C. An aliquot of 10 mL was titrated using 0.1% 2,6-dichlorophenolindophenol to obtain a pink color. Ascorbic acid concentration was computed and reported as g of AA per kg of fresh weight (g kg^−1^ AA).

#### 4.4.4. Polyphenol, Flavonoid, Anthocyanin, and Antioxidant Activity

Strawberry fruit tissue (1 g) was milled with 10 mL of methanol 50% (*v*/*v*) for 30 s. The extract was then ultra-sonicated for 30 min. Next, samples were placed in a shaker for 1 h at 200 rpm. The extract was centrifuged (Sigma 3-18K, Sigma Laboratory Centrifuge, Osterode am Harz, Germany) at 4000× *g* for 15 min at 4 °C. The supernatant was recovered and used for the analysis of total phenol content, total flavonoid content, and total antioxidant capacity (FRAP, ABTS).

The content of polyphenols was determined by employing the Folin-Ciocalteu method at 755 nm, according to Marinou et al. [62]. Crude extract (0.05 mL) was added to dH_2_O (final volume 1.625 mL) and mixed thoroughly with 0.125 mL of Folin-Ciocalteu reagent. The reaction was left to stand for 5 min, followed by the addition of 1.25 mL of 7% (*w*/*v*) Na_2_CO_3_ (final reaction volume 3 mL). The mixture was allowed to stand for a further 60 min at room temperature in the dark. The absorbance was measured at 755 nm using a spectrophotometer (Multiskan GO, Thermo Fisher Scientific Oy, Finland). The total phenol content was computed through a calibration curve of gallic acid, and the results were reported as g of gallic acid equivalents per kg of fresh weight (g kg^−1^ GAE).

The total flavonoids content of crude extract was determined using the aluminum chloride method [63]. The fruit extract (0.25 mL) was mixed with 1.525 mL of dH_2_O and 0.075 mL of 5% (*w*/*v*) NaNO_2_ and left to stand for 5 min. Then, 0.15 mL of AlCl_3_ 10% (*w*/*v*) was added to the mixture and the reaction was incubated for 5 min. Afterwards, 0.5 mL of 4% (*w*/*v*) NaOH was added to the mixture (total volume achieved 2.5 mL). The absorbance was measured at 510 nm. The result was reported as g of rutin per kg of fresh weight (g kg^−1^ rutin), using a calibration curve of prepared rutin dilutions.

The ability of the extract to reduce Fe^3+^ to Fe^2+^ and the formation of the blue colored [Fe (TPTZ)_2_]^2+^ complex was determined based on the following (FRAP) method from Chrysargyris et al. [64]. The FRAP reaction solution was prepared by combining 0.04 mL sample with 0.3M CH_3_COONa (pH = 3.6), 10 mM Tripyridil-s-triazine (TPTZ) and 20 mM FeCl_3_. A standard curve was also prepared using known (±)-6-hydroxy-2,5,7,8-tetramethylchromane-2-carboxylic acid (trolox) concentrations. The absorbance was measured spectrophotometrically at 593 nm after incubation at 37 °C for 4 min; the results were reported as g of trolox per kg of fresh weight (g kg^−1^ trolox).

The antioxidant capacity of strawberry fruit in the reaction with the ABTS radical cation was assessed according to the method outlined by Wojdyło et al. [65]. Briefly, the procedure was performed by mixing the ABTS solution 7 mM with 0.01 mL of sample. The mixture was incubated for 6 min at RT. A standard curve was prepared using known trolox concentrations. The absorbance at 734 nm was measured spectrophotometrically and the results were reported as g of trolox per kg of fresh weight (g kg^−1^ trolox).

The pH-differential method was used to determine the total anthocyanin content, using two buffers systems [66]. Strawberry tissue (2 g) was homogenized with 15 mL of methanol: dH_2_O:HCl (70:29:1, *v*/*v*/*v*). The homogenized extract was then centrifuged at 4000× *g* for 5 min at 4 °C. Next, the prepared extract was mixed with corresponding buffers (0.025 M KCl, pH = 1 and 0.4 M CH_3_COONa, pH = 4.5). After 15 min, the absorbance of each solution was measured at 520 and 700 nm using a spectrophotometer and calculated as g of cyanidin 3-glucoside equivalents per kg of fresh weight (g kg^−1^ cyn-3-glu).

### 4.5. Statistical Analysis

Analysis of variance (ANOVA) was used to analyze the data with the SPSS software (IBM SPSS version 22, Armonk, New York, NY, USA). Mean comparisons were made by Tukey’s multiple range test. Significance was defined at *p* ≤ 0.05.

## 5. Conclusions

This research investigated the preharvest effectiveness of commercial products based on natural compounds, i.e., chitosan, phosphoric acid plus micronutrients, and sweet orange EO, on strawberry postharvest decay and physiological qualitative parameters. Our results showed the greenhouse preharvest application of chitosan was effective against gray mold during shelf life. At the same time, the application of this compound maintained excellent qualitative parameters (i.e., color, respiration rate and TA) in the fruit. Our research suggests that this compound may be one of the most important natural compounds in related research and could be used for further investigation at the commercial level.

## Figures and Tables

**Table 1:** McKinney index of gray mold recorded on strawberries treated with commercial natural compound formulations. The fruit were kept for 7 d at 4 ± 1 °C, followed by 5 d shelf life at 20 °C and 95–98% RH. Different letters indicate that the values are significantly different according to a Tukey’s test (*p* ≤ 0.05).

Treatment	McKinney’s Index
	(%)
Control	55.71 ± 15.48 a
Chitosan	36.19 ± 9.11 b
Phosphoric acid plus micronutrients (PA+MN)	49.05 ± 13.57 ab
Sweet orange EO	49.52 ± 6.51 ab

**Table 2:** Effect of foliar spray of chitosan, phosphoric acid plus micronutrients (PA+MN), and sweet orange EO on strawberry fruit quality after harvest. Values (*n* = 6 for color measurements; *n* = 3 for others quality attributes) in rows followed by the same letter are not significantly different; *p* ≤ 0.05.

Quality Attributes	Control	Chitosan	PA+NM	Sweet Orange EO
Fresh weight (g)	19.89 ± 1.16 a	10.75 ± 2.94 b	14.50 ± 2.37 ab	17.44 ± 3.79 ab
Color L*	35.98 ± 1.55 a	39.01 ± 4.80 a	39.66 ± 3.75 a	38.4 ± 3.43 a
Color a*	34.45 ± 1.89 a	34.46 ± 4.08 a	32.00 ± 4.38 a	31.07 ± 3.21 a
Color b*	19.12 ± 2.74 a	33.70 ± 19.45 a	28.14 ± 17.21 a	22.00 ± 3.10 a
Chroma	39.48 ± 2.62 a	50.65 ± 17.09 a	44.24 ± 14.78 a	38.15 ± 3.91 a
Hue	0.50 ± 0.05 a	0.67 ± 0.18 a	0.65 ± 0.17 a	0.61 ± 0.06 a
WI	24.72 ± 1.62 a	19.15 ± 13.45 a	24.02 ± 10.03 a	27.23 ± 1.37 a
Color index	51.76 ± 7.79 a	38.12 ± 14.93 a	36.19 ± 12.02 a	38.23 ± 7.14 a
Respiration (mL kg^−1^ h^−1^ CO_2_)	26.75 ± 8.22 a	32.84 ± 6.80 a	29.31 ± 10.88 a	35.69 ± 15.84 a
Ethylene (mL kg^−1^ h^−1^)	19.40 ± 11.98 a	26.79 ± 16.63 a	37.55 ± 24.42 a	23.10 ± 19.22 a
Texture (N)	1.74 ± 0.60 a	2.11 ± 1.06 a	2.74 ± 0.68 a	2.88 ± 1.17 a
TSS (%)	8.06 ± 0.93 a	6.26 ± 1.97 a	7.90 ± 1.28 a	9.03 ± 1.60 a
TA (%)	0.93 ± 0.12 a	0.64 ± 0.09 b	0.81 ± 0.02 ab	0.93 ± 0.07 a
Ascorbic acid (g kg^−1^ AA)	1.40 ± 0.21 a	0.89 ± 0.13 b	0.79 ± 0.20 b	0.82 ± 0.14 b
Anthocyanins (g kg^−1^ cyn-3-glu)	0.03 ± 0.01 a	0.04 ± 0.03 a	0.02 ± 0.01 a	0.03 ± 0.01 a
Phenol (g kg^−1^ GAE)	2.56 ± 0.47 a	2.66 ± 0.12 a	2.92 ± 0.21 a	3.37 ± 0.48 a
FRAP (g kg^−1^ trolox)	3.53 ± 0.79 b	3.89 ± 0.97 ab	3.78 ± 0.50 ab	5.14 ± 0.65 a
ABTS (g kg^−1^ trolox)	10.34 ± 1.67 a	10.51 ± 1.03 a	7.18 ± 0.46 b	7.46 ± 0.66 b
Flavonoids (g kg^−1^ rutin)	0.66 ± 0.59 c	0.80 ± 0.20 bc	1.07 ± 0.69 ab	1.25 ± 0.21 a

## Data Availability

Not applicable.

## Supplementary Materials

The following supporting information can be downloaded at: https://www.mdpi.com/article/10.3390/ijms232415472/s1, Figure S1: Infection of postharvest gray mold on strawberries.
