# Supplementary figures and images for: Preharvest Application of Commercial Products Based on Chitosan, Phosphoric Acid Plus Micronutrients, and Orange Essential Oil on Postharvest Quality and Gray Mold Infections of Strawberry
Source: Int J Mol Sci. 2022 Dec 7;23(24):15472. doi: 10.3390/ijms232415472 (PMC9779177; doi:10.3390/ijms232415472)

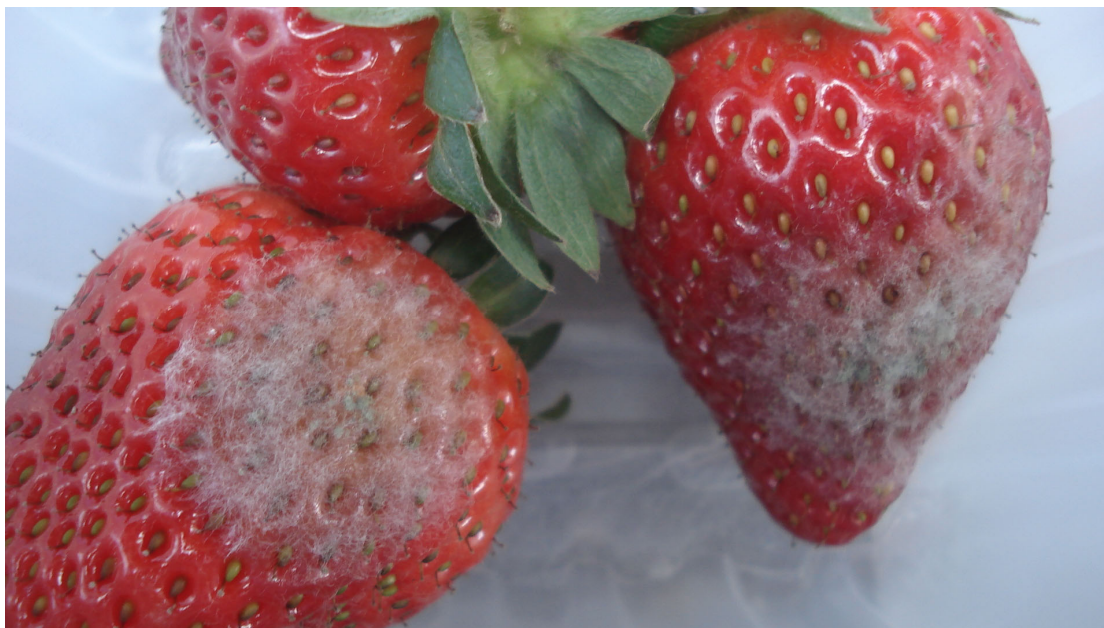

**Figure S1.** Infection of postharvest gray mold on strawberries.

Supplement: Supplementary file 1 [file ijms-23-15472-s001.zip › ijms-2019849-supplementary.pdf]
